# SPARC expression by cerebral microvascular endothelial cells in vitro and its influence on blood-brain barrier properties

**DOI:** 10.1186/s12974-016-0657-9

**Published:** 2016-08-31

**Authors:** Samir Alkabie, Jayasree Basivireddy, Lixin Zhou, Jane Roskams, Peter Rieckmann, Jacqueline A. Quandt

**Affiliations:** 1 Department of Pathology and Laboratory Medicine, University of British Columbia, Vancouver, BC Canada; 2 Department of Zoology, University of British Columbia, Vancouver, BC Canada; 3 Department of Medicine, Division of Neurology, University of British Columbia, Vancouver, BC Canada; 4 Sozialstiftung Bamberg, Klinikum am Bruderwald, Neurologische Klinik, Buger Str. 80, Bamberg, 96049 Germany

## Abstract

**Background:**

SPARC (secreted protein acidic and rich in cysteine) is a nonstructural, cell-matrix modulating protein involved in angiogenesis and endothelial barrier function, yet its potential role in cerebrovascular development, inflammation, and repair in the central nervous system (CNS) remains undetermined.

**Methods:**

This study examines SPARC expression in cultured human cerebral microvascular endothelial cells (hCMEC/D3)—an in vitro model of the blood-brain barrier (BBB)—as they transition between proliferative and barrier phenotypes and encounter pro-inflammatory stimuli. SPARC protein levels were quantified by Western blotting and immunocytochemistry and messenger RNA (mRNA) by RT-PCR.

**Results:**

Constitutive SPARC expression by proliferating hCMEC/D3s is reduced as cells mature and establish a confluent monolayer. SPARC expression positively correlated with the proliferation marker Ki-67 suggesting a role for SPARC in cerebrovascular development. The pro-inflammatory molecules tumor necrosis factor-α (TNF-α) and endotoxin lipopolysaccharide (LPS) increased SPARC expression in cerebral endothelia. Interferon gamma (IFN-γ) abrogated SPARC induction observed with TNF-α alone. Barrier function assays show recombinant human (rh)-SPARC increased paracellular permeability and decreased transendothelial electrical resistance (TEER). This was paralleled by reduced zonula occludens-1 (ZO-1) and occludin expression in hCMEC/D3s exposed to rh-SPARC (1–10 μg/ml) compared with cells in media containing a physiological dose of SPARC.

**Conclusions:**

Together, these findings define a role for SPARC in influencing cerebral microvascular properties and function during development and inflammation at the BBB such that it may mediate processes of CNS inflammation and repair.

**Electronic supplementary material:**

The online version of this article (doi:10.1186/s12974-016-0657-9) contains supplementary material, which is available to authorized users.

## Background

Endothelial cells play an essential role in normal homeostasis of the central nervous system (CNS). In healthy individuals, microvessels throughout most of the CNS possess a luminal monolayer of tightly apposed endothelial cells situated between the blood and brain parenchyma comprising together with adjacent astrocytes the blood-brain barrier (BBB) [1]. Cerebral endothelial cells are crucial for normal neurological function as they constitute both a physical “barrier” which limits molecular and cellular exchange between blood and brain compartments and a “fence” which maintains polarity of transporters responsible for delivery of essential nutrients and removal of potentially harmful toxins [2]. These CNS endothelia derive a low permeability barrier due to interendothelial tight junctions (TJ) occludin and claudin proteins as well as junction associated submembranous adaptor proteins such as zonula occludens (ZO)-1 [3]. Several studies show membrane localization of tight junction proteins are the morphological correlate of BBB integrity and tightness [4, 5]. Barrier disruption secondary to tight junction dysregulation results from reduced endovascular flow [6], hypoxia/ischemia [7], and inflammatory cytokines such as tumor necrosis factor-α (TNF-α) [8] and vascular endothelial-derived growth factor (VEGF) [9]. Several CNS diseases including neoplasia, hereditary vascular malformation, trauma, and chronic inflammatory and neurodegenerative diseases such as multiple sclerosis (MS) feature characteristics of BBB breakdown [10, 11]. Characterizing factors able to influence BBB integrity and aspects of vascular remodeling during CNS inflammation may identify key molecules with both physiological and perhaps pathological roles in disease.

BBB cytoarchitecture and response to stimuli are often examined in a simplified treatment and effect system comprised of in vitro cultures of endothelial cells established from cerebral microvessels. These cells recapitulate in vivo BBB characteristics such as expression of specific endothelial markers (i.e., CD31 and VE-cadherin), BBB transporters (i.e., GLUT-1, P-glycoprotein, transferrin), and tight junction markers (i.e., ZO-1 and occludin) and form a monolayer with low paracellular permeability and high transendothelial electrical resistance (TEER) consistent with the presence of membrane-associated tight junctions [12–14]. In the present study, we use a well-characterized in vitro model of the BBB consisting of immortalized human cerebral microvascular endothelial cells (hCMEC/D3) that express and appropriately localize important BBB proteins characteristic of their in vivo counterparts [15, 16].

SPARC (secreted protein acidic and rich in cysteine) is a matricellular cell-matrix modulating protein involved in angiogenesis [17, 18] and endothelial barrier function [19]. Many cell types including endothelia, fibroblasts, and macrophages constitutively express SPARC and up-regulate its expression in tissue regions undergoing high rates of remodeling, repair, and proliferation [20]. SPARC is typically enriched where new blood vessels are being formed, as evidenced using an in vivo chorioallantoic membrane (CAM) model of angiogenesis [21]. In the CNS, SPARC is highly expressed in developing blood vessels at early stages of development and down-regulated with developmental maturity (Roskams Lab, unpublished observations). This spatiotemporal pattern of SPARC expression is consistent with the role for SPARC in angiogenesis and BBB establishment [22, 23]. Normal physiological levels of SPARC in healthy individuals (0.1–0.8 μg/ml in plasma) are increased in neoplastic and inflammatory conditions (1.5–10 μg/ml in plasma) [24–26]. Increased SPARC secretion has been associated with carcinoma [27] and other tumors [28] such as gliomas [29], as well as inflammatory renal disease [30, 31], and scleroderma characterized by vascular dysfunction, autoantibody production, and tissue fibrosis [32].

SPARC may play a role in mediating the CNS response to injury and repair given enhanced expression in in vivo models of CNS damage and repair. In an in vivo cortical wound model, SPARC messenger RNA (mRNA) was abundantly expressed in developing blood vessels proximal to the wound edge days 3 to 10 post-injury, suggesting its involvement in the vascular response to CNS injury and cerebrovascular ischemia [22, 33]. Furthermore, SPARC may be associated with neurological recovery following CNS injury. Proteomic screens of murine lamina propria olfactory ensheathing cell (LP-OEC)-conditioned media identified SPARC as the key secreted protein supporting neural tissue repair after damage, capable of promoting spinal cord repair by limiting gliotic scar and cavity formation, stimulating axonal outgrowth and directing angiogenesis [34]. SPARC has been shown to promote Schwann cell-mediated neurite outgrowth in vivo and in vitro [34]. Moreover, SPARC-null OECs transplanted into contused rat spinal cord reduced outgrowth of specific subsets of sensory and supraspinal axons and impaired immune response to injury, suggesting its role in neural regenerative processes and the neuroimmune response to CNS injury [34].

Altogether, its potential roles in modulating angiogenic and barrier parameters of vascular development and repair [19, 23, 35, 36], its expression and influence on neural regeneration after CNS injury [33, 34], and its influence on the profile and extent of immune infiltration [37, 38] make SPARC a molecule of particular interest in chronic neuroinflammatory pathologies such as MS.

## Methods

### Reagents and antibodies

Recombinant human (rh)-SPARC (R&D Systems, Minneapolis, MN), TNF-α (Invitrogen, Camarillo, CA), interferon gamma (IFN-γ), (Invitrogen, Camarillo, CA), and lipopolysaccharide (LPS) (Sigma-Aldrich, St. Louis, MO) were obtained commercially. Antibodies for immunoblotting and immunocytochemistry included monoclonal mouse anti-human SPARC IgG_1_ (2.5 μg/ml; Haematologic Technologies, Essex Junction, VT), polyclonal rabbit anti-ZO-1 IgG (1 μg/ml; Invitrogen), monoclonal mouse anti-ZO-1 IgG_1_ (2.0 μg/ml; Invitrogen), monoclonal mouse anti-occludin IgG_1_ (0.5 μg/ml; Zymed, Carlsbad, CA), monoclonal mouse anti-Ki-67 IgG_1_ (1:100; Millipore, Billerica, MA), and monoclonal mouse anti-human glyceraldehyde-3-phosphate dehydrogenase IgG_1_ (GAPDH, 1:10,000; Santa Cruz Biotechnology, Santa Cruz, CA). Secondary antibodies included Alexa Fluor 568 goat anti-mouse IgG (1:200; Invitrogen); Alexa Fluor 488 goat anti-rabbit IgG (1:200; Invitrogen); and horseradish peroxidase (HRP)-conjugated goat anti-mouse IgG (1:5000; Jackson ImmunoResearch, Burlington, ON). Isotype-matched control abs included mouse IgG_1_ (2.5 μg/ml; Invitrogen) and rabbit IgG (1 μg/ml; Invitrogen).

### Cell culture

The hCMEC/D3 line was generously provided by Drs. B. Weksler, I. Romero, and P-O. Couraud (Cochin Institute, Paris). The cell line was established as described in Weksler et al. 2005. hCMEC/D3s were cultured in complete media comprised of EBM-2 media (Lonza, Walkersville, MD) supplemented with 5 % fetal bovine serum (FBS) (PAA Laboratories, Etobicoke, ON), 1 % penicillin-streptomycin, 1.4 μM hydrocortisone, 5 μg/ml L-ascorbic acid, 10 mM HEPES (all from Sigma), 1:100 chemically defined lipid concentrate (Invitrogen-Gibco), and 1 ng/ml basic fibroblast growth factor (bFGF) (Invitrogen-Gibco, Grand Island, NY) at 37 °C with 5 % CO_2_, 95 % air, and saturated humidity. The media was replenished every 2 to 3 days. For immunoblotting and reverse transcription polymerase chain reaction (RT-PCR), the cells were seeded at 1.2 × 10E4 cells/cm^2^ on 25 cm^2^ flasks and six-well plates (Corning, Corning, NY) coated with type I rat tail collagen (150 μg/ml; Sigma). For immunocytochemistry, the cells were grown on collagen type I-coated glass coverslips or collagenous membranes in a transwell configuration (MP Biomedicals, Solon, OH). hCMEC/D3 cells formed confluent, contact-inhibited monolayers, and were used consistently between passages (p)28 and 35.

### Growth and development of hCMEC/D3s

To test if SPARC expression changed under varied serum conditions, confluent hCMEC/D3 cultures (p30–32) were grown in complete growth media and replenished with fresh medium containing either no FBS, 1 % FBS, 5 % FBS, or 0.1 % bovine serum albumin (BSA) (Sigma) in place of FBS. hCMEC/D3 growth was assessed for culture confluency where cells were approximately 20–30 % confluent after 1 day in culture (DIC), 50–70 % confluent after 4 DIC, and confluent after 6–7 DIC. Protein and mRNA was extracted from cultures at subconfluent and confluent growth stages and subjected to immunoblotting and semi-quantitative RT-PCR. hCMEC/D3 cultures grown on collagen membranes were fixed in 4 % paraformaldehyde (PFA)-PBS when subconfluent (50–70 % confluent) and confluent, before being probed with monoclonal antibodies against SPARC, ZO-1, and Ki-67 for immunocytochemical analysis.

### hCMEC/D3 exposure to SPARC or inflammatory mediators

hCMEC/D3s cultured on collagen type I-coated six-well plates until confluent were treated 1 day later with 0.1, 1, 10 μg/ml rh-SPARC (R&D Systems) and TNF-α (200 U/ml) in fresh reduced serum (1 % FBS) complete media for 24 h. The protein levels of endothelial tight junctions were determined by Western blotting analysis. Confluent monolayers grown on collagen membrane inserts were replenished with complete media containing 10 or 100 U/ml TNF-α, 100, 200, or 500 U/ml IFN-γ, and 50 ng/ml LPS, alone and in combination. For cytokine treatment of cells on collagen membranes, media containing cytokine was added to the upper chamber.

### Barrier function assays

Barrier function was examined using TEER impedance measurements and transendothelial FITC-dextran (3 and 10 kDa) diffusion assays. hCMEC/D3 (p30–32) were grown on transwell polyester membrane inserts (0.4-μm porosity and 1.12 cm^2^ surface area, Corning Costar #3460) in serum-reduced (1 % FBS) complete media until 2 days post-confluence, and the measured TEER had stabilized. Media was replenished into both upper (luminal) and lower (abluminal) chambers; however, cytokine- and SPARC-containing media were only applied into the upper chamber. TEER was measured daily by EVOM-2 ohm meter using ENDOHM-12 chamber and STX2 chopstick electrodes (World-Precision Instruments, Sarasota, FL). Resistance of blank filters without cells was subtracted from those with cells, and these values were multiplied by the surface area of the inserts to get the final resistance (Ω·cm^2^).

Endothelial monolayer permeability was studied by measuring fluorescently labeled dextran diffusion through a confluent monolayer of hCMEC/D3 cells grown on transwell inserts. These studies were performed by adding 50 μg/ml dextran (3 or 10 kDa) tagged with Alexa Fluor 488 (Molecular probes, Eugene, OR) to the luminal chamber of a transwell insert and sampling (10 μl/sample) from the abluminal chamber at 30, 60, 120, and 180 min into a 96-well plate, an equal volume of fresh medium replaced each time. Dextran fluorescence in the samples was measured by a microplate fluorometer (Floroskan Ascent 374, ThermoScientific, Hudson, NH) at 485 nm (excitation) and 525 nm (emission). Raw values from dextran fluorescence were converted to concentrations using a standard curve and slope of the linear regression line. Permeability coefficient was calculated as described previously [4].

### Cell lysis and Western blotting

To lyse hCMEC/D3 cells grown on six-well plates, the cells were first washed twice with cold PBS, then incubated in situ on ice for 10 min in 125 μl of ice-cold NP-lysis buffer (50 mM Tris-HCl, 150 mM NaCl, 5 mM EDTA, and 1 % NP-40, adjusted to pH 8.0) with fresh protease inhibitor and phosphatase inhibitor cocktails added before use (Roche, Laval, QC). The cells were then scraped and collected into tubes, triturated on ice through a 28-Gauge needle five times and centrifuged at 13,500 rpm for 12 min at 4 °C. The supernatant was collected as whole cell lysate and stored at −80 °C. hCMEC/D3 cell lysis for TJ protein analysis required stronger detergents, 125 μl of ice-cold radio-immunoprecipitate assay (RIPA) buffer containing 20 mM Tris-HCl, 150 mM NaCl, 2 mM EDTA, 1 % NP-40, 0.1 % SDS, and 0.5 % sodium deoxycholate.

Protein concentrations were determined by bicinchoninic acid (BCA) protein assay kit (Sigma). Protein estimations based on the BSA standard absorbance curve. Lysates (20–40 μg) were diluted 1:1 in reducing 2× Laemmli sample buffer (Bio-Rad, CA) containing 0.05 % β-mercaptoethanol and heated at 100 °C for 5 min. The samples were subjected to sodium dodecyl sulfate-polyacrylamide gel electrophoresis (SDS-PAGE: 6 % gel for ZO-1, 12 % gel for SPARC, 15 % gel for occludin and claudin-5) for 1.5 h at 80–100 V and transferred to nitrocellulose membranes in wet transfer buffer (25 mM Tris, 192 mM glycine, 20 % methanol) at 90 mA overnight at 4 °C. Immunoblots for SPARC, claudin-5, occludin, and GAPDH were blocked in 5 % skim milk in Tris-buffered saline (TBS: 1.37 M NaCl, 27 mM KCl, 0.25 M Tris, adjusted to pH 7.4) for 1 h then incubated in primary antibody diluted in 2 % skim milk-TBST (TBS + 0.01 % Tween 20 (TW20)) overnight at 4 °C (or 2 h at RT for GAPDH). After washing, HRP-conjugated secondary antibodies diluted in 2 % skim milk-TBST (TBS + 0.01 % TW20) were added and then again washed in TBST. Immunoblots for detection of ZO-1 were blocked in 1 % BSA-TBST (TBS + 0.05 % TW20) for 4 h at RT and then incubated in monoclonal anti-ZO-1 primary antibody diluted in TBS overnight at 4 °C followed by washing in TBST and incubation with secondary antibody. All immunoblots were developed in enhanced chemiluminescence (ECL) substrate (ThermoFisher Scientific, Bothell, WA). Band signals were detected using a Versadoc imaging system (Bio-Rad Laboratories), and band densities were quantified by ImageJ 1.42i software (National Institutes of Health, Bethesda, MD).

### Semi-quantitative RT-PCR

Total RNA was extracted from cell cultures using RNeasy Mini Kit (QIAGEN Science, Toronto, ON) according to the manufacturer’s protocol. Complementary DNA (cDNA) was synthesized by High Capacity RNA-to-cDNA Master Mix Kit (Applied Biosystems, Foster City, CA). Relative quantification of PCR reactions were performed with Platinum PCR SuperMix (Invitrogen) using the following primer sequences for SPARC transcript amplification 5′-AGTGCACCCTGGAGGGCACC-3′ (Forward); 5′-TGCTTGATGCCGAAGCAGCC-3′ (Reverse) and the following primers for the GAPDH housekeeping gene 5′-AAGGCTGGGGCTCATTTGCAG-3′ (Forward); 5′-CTGCTTCACCACCTTCTTGATG-3′ (Reverse). A semi-quantitative analysis of mRNA levels was carried out using scans with the Bio-Rad Gel Doc UV system (Bio-Rad Laboratories, Hercules, CA) and differences in SPARC and GAPDH expression were calculated by Image Lab software (Bio-Rad Laboratories).

### Immunocytochemistry

Following co-incubations, cultures were washed twice with warm (37 °C) PBS and fixed at RT in 4 % PFA-PBS for 10 min. Cultures were directly stored in PBS-0.01 % sodium azide at 4 °C until stained. For staining, cultures were washed twice with PBS then incubated for 10 min in permeabilization-blocking buffer (0.1 % Triton X-100 and 4 % normal goat serum (NGS) in PBS). Cultures were incubated twice for 10 min in blocking buffer (4 % NGS-PBS) then incubated with primary antibody diluted in blocking buffer for 1 h at RT or overnight at 4 °C. After primary antibody incubation, cultures were washed three times for 5 min with PBS then incubated in specific secondary antibody in blocking buffer for 50 min in the dark. Cultures were washed twice in PBS, stained with DAPI nuclear stain (1:10,000) in PBS for 5 min, and again washed three times in PBS. Membranes were excised from discs using a scalpel, drained of excess PBS, and embedded in 10 μl of ProLong Gold anti-fade reagent (Invitrogen) underneath a glass coverslip. Cultures on the glass coverslips were mounted cell side down on 10 μl of ProLong Gold.

### Image acquisition and analysis

Distribution analysis was performed in a blinded fashion where cells were distinguished and coded numerically on a DAPI image to avoid duplication and evaluated for level of SPARC expression. SPARC immunoreactivity levels were assigned to individual cells according to the classification scheme and representative images in Table 1. Fluorescence images were captured with an Axioplan 2 imaging epifluorescent microscope (Zeiss, Jena, Germany) and Axiovision 4 software (Zeiss). Confocal micrographs were captured with an Olympus Fluoview 1000 laser scanning confocal microscope with Nomarski optics and FV1000 Fluoview software (Olympus Corporation, Tokyo Japan). Analysis was performed using Adobe Photoshop extended CS3 version 10.0 or ImageJ 1.42i software (NIH) or assessed by SPARC immunoreactivity scale. Adobe Photoshop measurements of nuclear Ki-67 intensity were performed by outlining individual nuclei on a DAPI/blue filter image using the “quick selection tool”, and measuring Ki-67/green filter mean pixel intensity (MPI)—the average intensity of all pixels within the DAPI-stained/delineated nuclear regions. Ki-67 positivity was relative to a threshold determined by blindly screening three images of confluent and subconfluent images each and denoting those cells negative for Ki-67. A threshold limit was set by averaging the Ki-67 MPI of those nuclei visually deemed Ki-67 negative (*n* = 59) plus three standard deviations. Ki-67-positive nuclei were those exceeding this pre-defined Ki-67 threshold. Quantification for SPARC immunofluorescence was performed either by regional analysis, where regions for analysis were demarcated by thresholding the cell edge such that only the cell-covered surface area of a field/image was assessed for MPI using ImageJ or assessed based on the SPARC immunoreactivity scale. Cell counting of DAPI-positive nuclei was performed using the ImageJ “analyze particles” tool and automated using ImageJ macroscript.

**Table 1:**
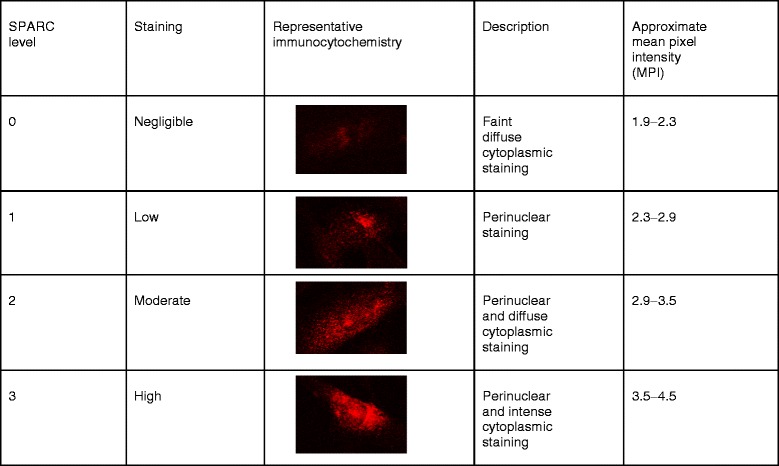
SPARC immunoreactivity assessment scale for hCMEC/D3 cells in culture

### Statistical analysis

GraphPad Prism version 5.01 (GraphPad Software, San Diego, CA) was used for graph synthesis and statistical analysis. Parametric data expressed as mean ± standard error of the mean (mean ± SEM) were analyzed by one-way analysis of variance (ANOVA) and Newman-Keul multiple comparison test. Non-parametric data expressed as mean ± standard deviation (mean ± SD) were analyzed by a Kruskall-Wallis and Dunn’s multiple comparison test. Mann-Whitney comparison was performed on data comparing two non-parametric groups/treatments. SPARC immunoblots and functional assays were analyzed by one-way ANOVA followed by a Tukey’s honestly significant difference (HSD) multiple comparisons analysis.

## Results

### The in vitro hCMEC/D3 model of the BBB

**Fig. 1:**
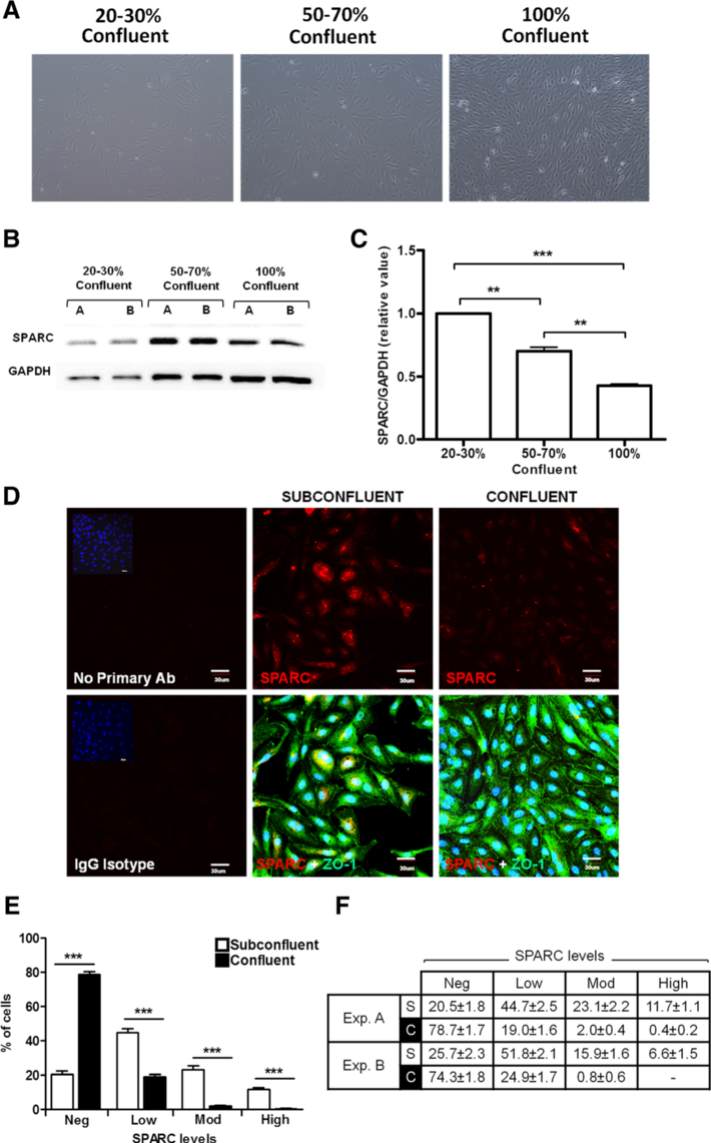
SPARC expression is associated with proliferation of immature hCMEC/D3 and decreased as cells mature and reach confluence. **a** Confluency and monolayer intactness were visually assessed by phase contrast microscopy. **b**, **c** hCMEC/D3 cultures 20–30, 50–70, and 100 % confluent were analyzed for SPARC protein expression by immunoblotting. Data is pooled from two independent experiments, *error bars* represent standard error of the mean (SEM) with statistical significance by one-way ANOVA and Tukey’s comparison test **P < 0.001; ***P < 0.0001. **d** Representative confocal micrograph images show greater perinuclear and cytoplasmic SPARC staining (*red*) in subconfluent cultures compared to confluent cultures. Subconfluent cultures exhibit discontinuous peripheral ZO-1 bands (*green*), whereas confluent cells were tightly apposed and organized into monolayers with continual ZO-1 bands at interendothelial borders. *Scale bar* = 30 μm. **e** The individual cells in confluent monolayers express less SPARC than the cells in subconfluent cultures. Levels of SPARC immunoreactivity (negligible, low, moderate, or high) were assigned according to Table 1 (Additional file 1: Figure S1). *Bars* represent the average of results from *n* = 18 and *n* = 10 images enumerated for low, medium, or high levels in subconfluent (*empty bar*) vs. confluent (*filled bar*) cultures in replicate wells. **f** Summary of individual cell staining classification from both Fig. 1e (*exp. A*) and a second experiment (*exp. B*) with similar findings for the distribution of cells from (*S*) subconfluent vs. (*C*) confluent cultures

hCMEC/D3 cells grown on collagen type I-coated surfaces had fusiform morphology and were tightly apposed forming monolayers with minimal overlap upon reaching confluence (Fig. 1a). The cells grown on collagen membrane inserts (primarily type I collagen with some type IV collagen) were comparable in morphology and growth rate to that of cells grown on type I collagen-coated flasks or filters and thus became our preferred culturing surface for immunocytochemistry experiments.

### SPARC expression during hCMEC/D3 growth and maturation

SPARC is known to be expressed in different tissues during remodeling and repair [20], where SPARC expression correlates with cell propagation. SPARC protein expression here was first quantified by Western blot on cells at 20–30, 50–70, and 100 % confluency (Fig. 1a). Immunoblotting and immunocytochemistry analysis showed subconfluent cultures had greater SPARC expression than confluent cultures. SPARC expression decreased with days in culture with greater than 50 % down-regulation as hCMEC/D3 established a confluent monolayer (Fig. 1b, c). These experiments provide evidence that SPARC is highly expressed in hCMEC/D3 cells during proliferative stages of growth and down-regulated as cells establish a monolayer and become contact inhibited.

To investigate SPARC expression during hCMEC/D3 growth and as cells develop an in vitro BBB phenotype, cultures were fixed when approximately 50–70 % confluent (subconfluent) and confluent then subjected to immunocytochemical staining. Confluency was visually assessed by phase contrast microscopy. Barrier phenotype was confirmed by peripheral localization of ZO-1, a marker of tight junction formation. In subconfluent cultures, cells exhibited perinuclear and dense cytoplasmic ZO-1 staining with ZO-1 rarely limited peripherally to cell borders (Fig. 1d). Conversely, confluent cells exhibited tightly apposed monolayers with minimal overlap (Fig. 1a, d) and ZO-1 bands delineating the cytoplasmic membrane in the majority of cells (Fig. 1d). Immunocytochemical analyses revealed that SPARC levels were heterogeneous among hMEC/D3 cells in culture. The intensity of SPARC expression (negligible, low, moderate, or high) in the subconfluent and confluent cultures was assessed on confocal images. The subconfluent cells predominantly exhibited low, moderate, and some high SPARC levels compared to the confluent cultures which were largely (~80 %) negligible while the remainder of cells were low in SPARC expression (Fig. 1e, f).

SPARC expression was also examined on high-resolution confocal micrographs by quantifying the global mean pixel intensity of the images. SPARC intensity was measured in subconfluent and confluent cultures within a 300 μm × 300 μm field on images pooled from replicate wells reported as relative mean pixel intensity ± standard deviation (MPI ± SD). SPARC intensity was greater in regions of subconfluent culture (3.85 ± 0.492) than regions of confluent culture (2.83 ± 0.426). Confocal micrographs revealed that subconfluent cultures with low and discontinuous ZO-1 staining at cell borders have greater SPARC immunoreactivity than confluent cultures which showed intense ZO-1 banding at cell-cell contacts (Fig. 1d) and minimal SPARC expression (Fig. 1d).

### SPARC expression correlates with the proliferation marker Ki-67

**Fig. 2:**
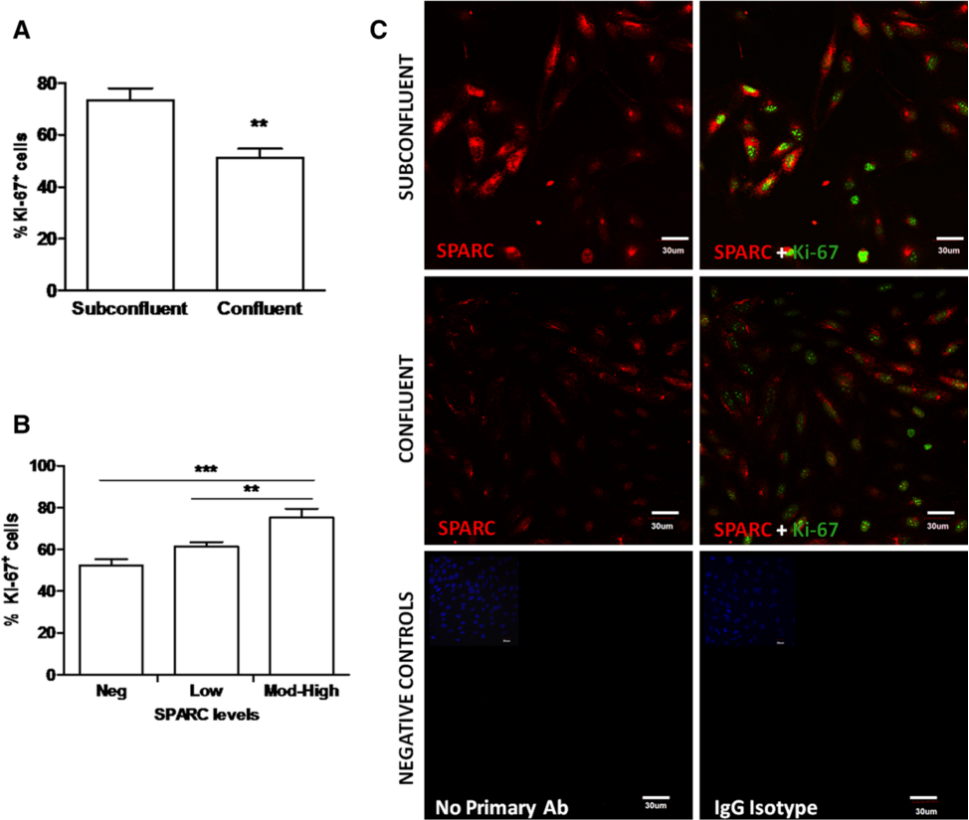
SPARC expression positively correlates with a marker of proliferation Ki-67. **a** Representative immunocytochemistry images of subconfluent hCMEC/D3 show greater SPARC expression and higher proportions of Ki-67-positive cell nuclei than confluent cultures. Images represent SPARC staining (*red*) alone (*upper panel*) or merged with Ki-67 staining (*green)* in the *lower panel*. Data represents one of two replicate experiments. *Scale bar* = 30 μm. **b** Percentage of cells positive for the proliferation marker Ki-67 in the subconfluent hCMEC/D3 is greater than that of the confluent cultures. Confluent hCMEC/D3 in two experiments had significantly (exp. A: *P* = 0.0143; exp. B: *P* = 0.0017) lower incidence of Ki-67-positive nuclei than the subconfluent cultures. *Bars* represent the average of results (20 images from triplicate subconfluent wells and 8 images from duplicate confluent wells) grown on collagen membranes ± standard deviation (SD). Mann-Whitney comparison test, **P < 0.001. **c** SPARC levels positively correlated with the percent of Ki-67-positive cell nuclei in subconfluent hCMEC/D3 cultures. Ki-67 staining is significantly different between groups classified according to increasing levels of SPARC staining from negligible to low through moderate and high combined (*P* < 0.0001). *Bars* represent the average of results from *n* = 20 images pooled from triplicate wells. *Error bars* indicate SEM. Data represents one of the two experiments with similar significant results. One-way ANOVA and Tukey’s multiple comparison test, **P < 0.001, ***P < 0.0001

The Ki-67 protein (also known as MKI67) is a cellular marker for proliferation present during all active phases of the cell cycle (G1, S, G2, and mitosis) but typically absent from resting cells (G0) [39, 40]. Consistent with a relatively quiescent versus proliferative state, cells from confluent cultures had a significantly reduced percentage of Ki-67-positive or bright cell nuclei than subconfluent cultures (Fig. 2a, *P* = 0.01). The immortalized nature of this cell line would be consistent with lower, often speckled or punctate, but not negligible incidence of Ki-67 staining in confluent cultures. To further examine associations between relative SPARC expression and Ki-67 reactivity in individual cells, we focused on subconfluent cells which had SPARC expression in all ranges of our classification scheme. Notably, cells in subconfluent cultures with negligible, low, or moderate to high levels of SPARC expression demonstrated significantly increasing frequencies of Ki-67-positive or bright cell nuclei (Fig. 2b; *P* < 0.0001). Representative images demonstrate that cells in confluent cultures were more likely to demonstrate colocalization of SPARC along with brighter Ki-67 staining; conversely, confluent cultures showed faint SPARC staining and fewer Ki-67 bright cells than observed in subconfluent hCMEC/D3 (Fig. 2c).

### SPARC expression is influenced by serum conditions

**Fig. 3:**
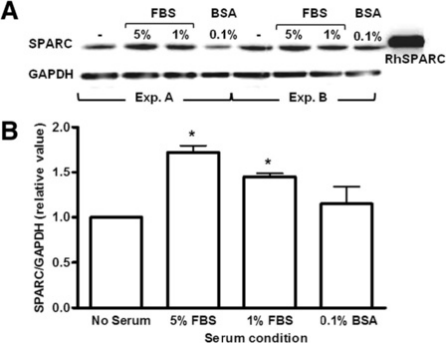
**a**, **b** Growth media serum concentration influences SPARC expression in confluent hCMEC/D3 cells. SPARC protein levels were quantified by western blot analysis of lysates derived from cultures propagated in media with varied serum concentration. Relative SPARC protein levels were normalized to GAPDH. SPARC detected in lysates resolved at the molecular weight observed for rh-SPARC (~43 kDa). *Bars* represent the average of four immunoblots, two independent experiments (exps. A and B) performed in duplicate. *Error bars* indicate SEM. One-way ANOVA and Tukey’s multiple comparison test, *P < 0.05

Serum has been defined as a potential stimulatory and mitogenic factor for cells in primary culture as well as cell lines in vitro [41]. The ability of hCMEC/D3 cultures to fare well in reduced serum allowed us to examine the effects of serum dose on SPARC expression. Five percent serum-supplemented media recommended for hCMEC/D3 cell line expansion produced the highest observed levels of SPARC protein. One percent serum also significantly increased SPARC compared to media lacking serum (Fig. 3a, b). Culturing cells in 0.1 % BSA resulted in SPARC expression more comparable to levels observed in serum-free cultures (Fig. 3a, b). These data clearly demonstrate that SPARC expression in hCMEC/D3 cells is influenced by serum concentration in culture media. To enhance our sensitivity to detect increases in SPARC expression secondary to inflammatory stimuli, subsequent treatment experiments were carried out in the presence of 1 % serum where constitutive SPARC expression was demonstrated to be low.

### Inflammatory mediators regulate SPARC expression in hCMEC/D3s

**Fig. 4:**
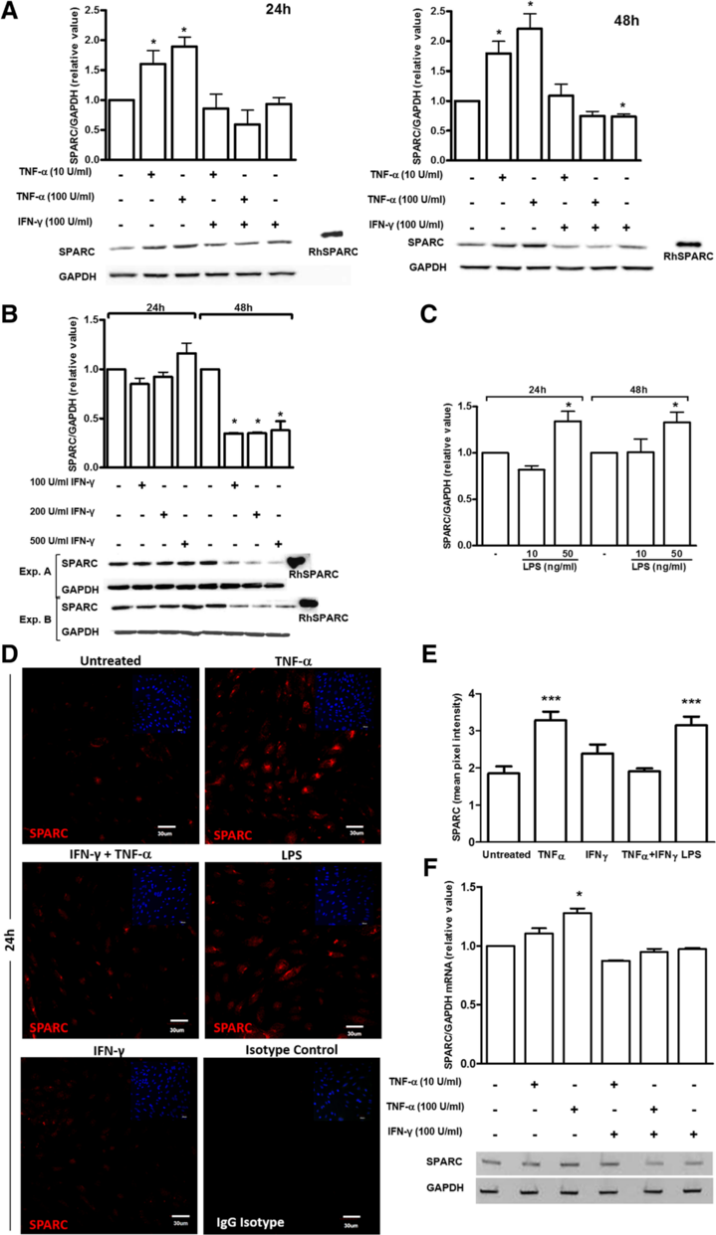
Inflammatory mediators differentially regulate SPARC expression in hCMEC/D3 monolayers. hCMEC/D3 were incubated for 24 and 48 h in replenished media with TNF-α, IFN-γ, or LPS, alone or in combination. **a** Compared to untreated/control conditions, 10 and 100 U/ml TNF-α treatment alone increased SPARC expression at 24 and 48 h. *Bars* represent the average relative value of blots from *n* = 3 independent experiments. *Error bars* indicate SEM. ANOVA and Tukey’s multiple comparison test, *P < 0.05. **b** The effect of varied concentrations of IFN-γ (100, 200, 500 U/ml) on SPARC expression in hCMEC/D3 cultures was assessed by Western blot analysis. **c** LPS treatment enhanced SPARC levels in a dose-dependent manner at 24 and 48 h by Western blotting. Pooled results from two biological replicates are shown. **d** The effect of inflammatory mediators on SPARC expression in confluent hCMEC/D3 cultures was assessed in parallel by quantitative immunocytochemistry with representative images shown and summary of findings shown **e**. SPARC expression is shown following 100 /ml TNF-α, 100 U/ml IFN-γ, or 25 ng/ml LPS alone or in combination. Quantification was pooled from 10 images of duplicate wells for each treatment. Untreated confluent cultures showed minimal SPARC staining at 24-h TNF-α and LPS treatment alone increased SPARC levels after 24 h (*P* < 0.0001). Data represents one of the two experiments with similar results; *error bars* represent SEM. One-way ANOVA and Tukey’s multiple comparison test vs. untreated controls, *P < 0.05; **P < 0.001; ***P < 0.0001. *Scale bar* = 30 μm. **f** RT-PCR was used to quantify SPARC mRNA. 100 U/ml TNF-α treatment significantly (*P* < 0.05) increased SPARC mRNA expression. *Bars* represent the average densitometric value from *n* = 4 experiments; *error bars* represent SEM. One-way ANOVA and Tukey’s multiple comparison test vs. untreated controls, *P < 0.05

Our in vitro approach allowed us to model the influence of specific inflammatory molecules on cell morphology and SPARC expression at the BBB. hCMEC/D3 cultures grown to confluence were incubated for 12, 24, and 48 h with TNF-α, IFN-γ, or LPS, alone or in combination. SPARC expression patterns were studied after treatment with pro-inflammatory cytokines TNF-α and IFN-γ, implicated in the pathogenesis of several CNS inflammatory disorders and the endotoxin LPS, a primary component of the bacterial cell wall known to stimulate the innate immune response to infection. Cell confluence and morphology was assessed by phase contrast and ZO-1 immunofluorescence. SPARC expression was quantified by immunofluorescence and immunoblotting. Untreated cultures showed tightly apposed cells with minimal overlap at the time of treatment and regular fusiform morphology throughout the treatment period. Treatment with pro-inflammatory mediators resulted in elongated morphology and whirling into circular patterns along with a disruption in peripheral ZO-1 staining (data not shown). SPARC immunoreactivity was minimal in untreated confluent cultures maintained in serum-reduced media (Fig. 4a, d) and significantly increased following exposure to 10 and 100 U/ml TNF-α for 24 to 48 h (Fig. 4a). 50 ng/ml LPS treatment induced SPARC levels to that observed for 100 U/ml TNF-α alone, while 10 ng/ml LPS had no effect (Fig. 4b, d). Interestingly, 100 U/ml TNF-α and 100 U/ml IFN-γ co-treatment abrogated SPARC induction otherwise observed for TNF-α alone at 24 and 48 h (Fig. 4a, d).

The modulatory effect of IFN-γ on SPARC expression in hCMEC/D3 cells was temporally delayed. Significant decreases in SPARC protein level were not observed until 48 h of IFN-γ treatment (Fig. 4c) However, the decrease in SPARC expression in response to a combination of IFN-γ (100 U/ml) and TNF-α (100 U/ml) was evident much earlier at 12 h (data not shown) and significant as early as 24 h. We further analyzed the effects of various concentrations of IFN-γ on SPARC protein level. To our surprise, higher concentrations of IFN-γ (200, 500 U/ml) did not significantly change SPARC expression at 24 h and was comparable to 100 U/ml at 48 h compared to untreated controls. Taken together, these results show that TNF-α alone or LPS consistently increased intracellular SPARC protein expression, while IFN-γ alone or in combination with TNF-α limits its enhancement in hCMEC/D3 cells. SPARC expression was also analyzed at the individual cell level (Fig. 4d) and across areas of confluent cells (Fig. 4e). SPARC expression as measured by mean pixel intensity was significantly increased in cells treated with TNF-α alone or with LPS alone, which correlated well with Western blot data. Treatment of hCMEC/D3 cultures with TNF-α did up-regulate SPARC mRNA expression in a dose-dependent manner where only 100 U/ml TNF-α reached significance in the time studied.

### Exogenous SPARC treatment increases cerebral endothelial barrier permeability

Secreted SPARC binds matrix components of the basement membrane and modulates cell-matrix interactions thought to affect endothelial barrier function [42]. Direct evidence suggests that SPARC increases transendothelial permeability of hCMEC/D3 monolayers and may play a role in modulating BBB permeability. To understand the role of exogenous SPARC on modulating BBB integrity, rh-SPARC was applied to hCMEC cultured on transwell inserts 1 day post-confluence. SPARC treatment was performed in serum-reduced media with 1 % FBS to limit endogenous SPARC production (compared to that observed with higher serum conditions). We assumed that a lower endogenous SPARC level might clarify the effect of exogenously supplied SPARC on permeability.

**Fig. 5:**
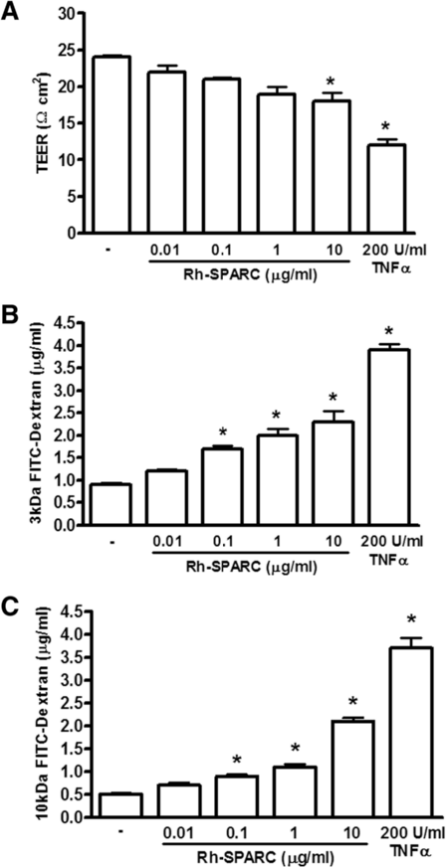
SPARC influences hCMEC/D3 barrier function measured by TEER and permeability. **a** Confluent hCMEC/D3 on transwell inserts were replenished with media containing rh-SPARC (0.01, 0.1, 1, 10 μg/ml) for 24 h then tested for TEER and transendothelial diffusion of FITC-labeled dextran (3 and 10 kDa). Compared to the untreated conditions, TEER measurements reflect a dose-dependent reduction in electrical impedance with increasing rh-SPARC concentration reaching significance with 1 μg/ml or greater rh-SPARC. Permeability assays using **b** 3 and **c** 10 kDa FITC-dextran revealed exposure of hCMEC/D3 to 0.1–10 μg/ml rh-SPARC enhanced permeability to both by 24 h of exposure. One-way ANOVA and Tukey’s multiple comparison test vs. untreated controls, *P < 0.05

TEER on hCMEC/D3 remained relatively low throughout this study. It is documented that hCMEC/D3 cultures have low TEER under static conditions [6, 16]. Baseline TEER recordings were between 20 and 25 Ω·cm^2^ by ENDOHM-12 chambers and 55–60 Ω·cm^2^ by STX2 chopstick recordings across untreated hCMEC/D3 monolayers. Application of rh-SPARC decreased TEER in a concentration-dependent manner (Fig. 5a). Significant TEER reduction was seen only at our greatest SPARC concentration tested of 10 μg/ml at 24 h but was still only about half of the TEER change observed following TNF-α (200 U/ml) treatment.

Cultures used to measure TEER were also used in FITC-dextran diffusion permeability assays. Microvascular cerebral endothelia partially comprise the BBB and form a functional barrier interface between blood and neural parenchyma regulating vital functions such as fluid-ion balance and essential nutrient delivery. Addition of exogenous SPARC increased the permeability of hCMEC/D3 monolayers as measured by increased FITC-dextran diffusion after 24 h. This increase was concentration dependent where more SPARC produced a greater increase in permeability to both 3- and 10-kDa dextrans (Fig. 5b). A dose of SPARC greater than 0.01 μg/ml SPARC was sufficient to increase the permeability of hCMEC/D3 monolayers. Our studies show that SPARC has direct effects on properties of the blood-brain barrier. Because TNF-α was also able to drive SPARC expression, we addressed whether or not SPARC may be contributing to TNF-α-mediated changes in permeability. Testing a variety of monoclonal as well as polyclonal antibodies for their potential to neutralize SPARC, we were not able to significantly influence TNF-α mediated changes in EC permeability detected by dextran diffusion (data not shown). In this regard, the primary TNF-α induced alterations in BBB permeability would seem to occur independent of SPARC.

### SPARC alters tight junction protein levels in hCMEC/D3s

To test the ability of SPARC to alter BBB-associated tight junction protein expression, hCMEC/D3 monolayers were treated with rh-SPARC (0.1, 1, 10 μg/ml) or TNF-α (200 U/ml), the latter being a well-characterized modulator of TJ expression in BBB endothelial cells, including hCMEC/D3 cells [16]. Phase contrast micrographs of TNF-α and SPARC-treated hCMEC/D3 monolayers showed cultures remained intact under all treatment conditions and retained regular fusiform morphology and apposition with minimal overcrowding and no apparent lifting or dissociation (data not shown). Previous studies of BBB integrity and TJ expression have described a barrier-promoting/protecting property for hydrocortisone [4]; hydrocortisone was included at doses recommended by the hCMEC/D3 providers based on publications testing its influence on barrier phenotype. Cell morphology and growth appeared similar in the presence or absence of hydrocortisone after 24 h (data not shown).

**Fig. 6:**
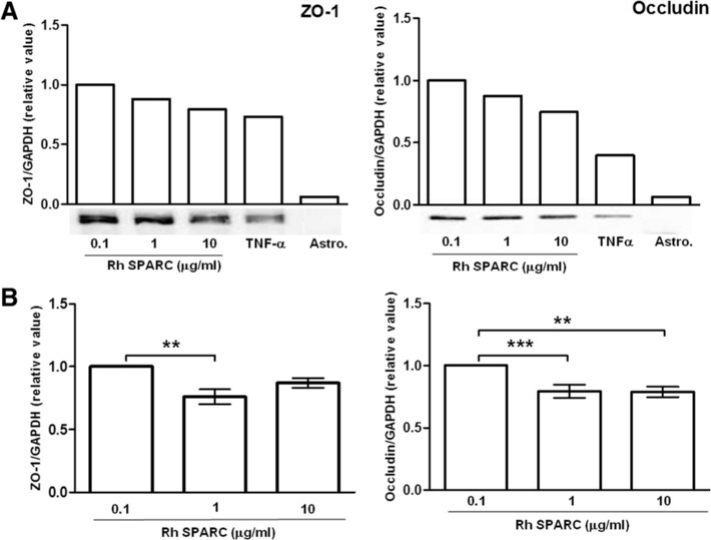
SPARC and TNF-α regulate hCMEC/D3 tight junction protein expression. **a** Confluent hCMEC/D3 cultures treated with SPARC (0.1, 1, 10 μg/ml) or TNF-α (200 U/ml) for 24 h were analyzed for tight junction (TJ) expression. TJ expression was quantified by immunoblot analysis of hCMEC/D3 lysates with the TJ-negative human astrocytoma cell line (Astro) ccf-sttg1 (Sigma) lysate as a negative control. A reduction in endothelial TJ expression was observed with increasing SPARC exposure above 1 μg/ml. **b** Increasing exogenous SPARC from 0.1 to 1 μg/ml lowered both ZO-1 (by 23 %) and occludin (by 20 %) expression. The *bars* represent the mean expression from 5 (ZO-1) and 4 (occludin) experiments, and *error bars* represent the SEM. **P < 0.001; ***P < 0.0001 by ANOVA and Student-Newman-Keuls multiple comparisons test

Addition of TNF-α decreased total expression of all TJ proteins studied (Fig. 6a). TNF-α treatment of hCMEC/D3s lowered the expression of ZO-1 (0.76, *P* = 0.013) and occludin (0.62, *P* = 0.044) compared to controls. The addition of hydrocortisone to the media with TNF-α returned ZO-1 expression to baseline levels, but was not effective at restoring occludin levels to that of untreated cells when in the presence of TNF-α (data not shown).

Our experiments revealed that hCMEC/D3 cells in culture produced low levels of SPARC even under resting conditions, suggesting that levels of SPARC in culture closely model the physiological levels of SPARC detected in the plasma and serum of healthy individuals (ranging from 0.1 to 0.5 μg/ml). In this regard, it is difficult to assess the physiological relevance of comparing cells cultured in “0” or “no SPARC” conditions which would exist immediately following media replacement.

Exogenous SPARC was applied at physiological levels of 0.1 μg/ml and at supraphysiological levels observed in individuals with various inflammatory disorders. Functional studies using FITC-dextran (3 and 10 kDa) diffusion and TEER showed that rh-SPARC treatment increases transendothelial permeability of hCMEC/D3 monolayers in a concentration-dependent manner (Fig. 5b, c), confirming barrier disruption in cerebral monolayers when exposed to SPARC in physiological (0.1 μg/ml) and supraphysiological (1–10 μg/ml) concentrations. Furthermore, we were also interested in whether SPARC altered TJ expression at the BBB. Increasing concentrations of exogenous SPARC from 0.1 to 1 μg/ml lowered both ZO-1 (0.768, *P* < 0.05) and occludin (0.796, *P* < 0.001) protein levels (Fig. 6b). Concentrations in the range of 1 to 10 μg/ml tended to decrease ZO-1 and occludin protein levels compared to baseline SPARC serum concentrations (approximately 0.1 μg/ml) (Fig. 6a, b). Representative immunoblots and normalized quantifications depict the trends observed (Fig. 6a).

## Discussion

### SPARC expression during cerebral endothelial growth and barrier establishment

This is to our knowledge the first study to examine SPARC expression and regulation in an in vitro BBB model. This report describes novel in vitro evidence that cerebral endothelia constitutively express SPARC during proliferative stages of growth and down-regulate it as cells form a monolayer and establish a BBB-like phenotype as assessed by Western blot and quantitative immunocytochemistry. This is consistent with in vivo observations where SPARC expression was enriched in developing cerebrovasculature yet diminished in mature vessels (Roskams lab, unpublished observations) [22, 23, 43].

hCMEC/D3 cells in our experiments were propagated in media containing various concentrations of serum which reproducibly correlated with SPARC expression. Cell cultures replenished for 24 h in media with increasing serum concentrations were found to express increased levels of SPARC. Serum is a known mitogen in this cell line used as a positive control and stimulus of cell growth and proliferation [41]. Our experiments define serum as a positive regulator of SPARC expression in cerebral endothelia during proliferative stages of growth.

We performed dual immunocytochemistry for SPARC and a proliferation marker Ki-67 to investigate the linkage between SPARC and endothelial proliferation—a vital parameter of angiogenesis and cerebrovascular response to injury. SPARC positively correlated with Ki-67 in subconfluent hCMEC/D3 cultures. Increased SPARC immunoreactivity was associated with a higher percentage of Ki-67-positive nuclei while confluent monolayers shared low SPARC and low Ki-67 levels, providing a direct correlation between SPARC expression and proliferation. Brain endothelial cells have been shown to co-express high levels of SPARC and the marker of proliferation PCNA (proliferating cell nuclear antigen) in developing but not mature brain capillaries [43]. SPARC produced by dividing subconfluent cerebral endothelia may promote focal adhesion disassembly and inhibit cell spreading mediating cell migration and proliferation during CNS angiogenesis [44].

Elevated SPARC mRNA and protein expression has been observed in endothelial cells involved in angiogenesis [18, 45]. Bovine aortic and human microvascular endothelial cells that spontaneously form tube-like vessel structures revealed that new vessel branches are enriched in SPARC and possess a high mitotic index. Confocal microscopy in an in vivo model of angiogenesis similarly showed intense SPARC staining in small-caliber, newly formed, blood vessels and negligible staining in larger developmentally mature vessels [17, 36]. In the heart, SPARC coincided with blood vessel neo-vascularization on aortic valves yet not normal avascular valves [46]. Notably, SPARC expression was upregulated 4.2-fold in bovine aortic endothelial cells and 10-fold in rat cerebral endothelial cell (RVEC) cultures that spontaneously organized into capillary tubes in vitro [17, 21, 36], suggesting a role for SPARC in “de novo” morphogenesis of new microvessel angioarchitecture (vasculogenesis) and the growth of new vessels from established ones (angiogenesis). Taken together, these data suggest SPARC is highly expressed in developmentally new blood vessels undergoing angiogenesis in vivo, consistent with our in vitro data showing that SPARC expression in human cerebral endothelia corresponds with proliferation and the initial steps of brain microvessel formation.

SPARC expression in vivo coincides with developing blood vessels during embryogenesis and postnatal development, but not in mature blood vessels of the normal adult CNS. To that effect, in situ hybridization showed SPARC mRNA levels enriched in migrating endothelia and pia-derived blood vessels of embryonic and postnatal tissue progressively down-regulated with maturity in adult cerebrovasculature [22, 23, 33, 47]. In the normal (unlesioned) adult CNS, SPARC protein expression is predominantly restricted to neurogenic subventricular zones, choroid plexus, actively surveying microglia, specialized radial glia (Bergmann and Müller glia), and astrocytes [23, 34]. These data together suggest SPARC may be expressed by cerebral endothelia in a spatiotemporal manner consistent with a role in CNS vascularization.

### Regulation of SPARC expression during inflammation

Human microvascular blood vessels express elevated levels of SPARC at sites of injury, infection, and neoplasia relative to healthy tissue [28, 33, 44, 48]. Expression of SPARC in adult tissue and plasma increases in response to various environmental stressors, including heat shock, heavy metal toxicity, endotoxin [49], angiogenesis [21, 36], and wound repair [20]. Using an in vitro model of the BBB, this study offers the first description of inflammatory regulation of SPARC expression in cerebral endothelia. In the present study, the hCMEC/D3 cells exposed to the prototypical injury response cytokine TNF-α and the endotoxin LPS increased SPARC levels compared to the untreated cells. Notably, IFN-γ combined with TNF-α abrogated the SPARC induction otherwise observed in response to TNF-α alone, suggesting cross-talk between TNF-α and IFN-γ that may negatively regulate levels of SPARC in response to inflammation at the BBB. Although the present study is the first mention of pro-inflammatory regulation of SPARC expression in cerebral endothelia, other CNS injury models and neoplastic syndromes have highlighted the association between inflammation and SPARC-enriched blood vessels [48, 50].

In the normal adult CNS, SPARC levels are low and expression is primarily restricted to astrocytes and microglia of synaptic rich regions but typically not blood vessels. That balance appears to shift during a number of inflammatory states and models. In a cortical wound model of CNS injury, SPARC mRNA was increased in blood vessels proximal to the wound [33]. During the peak phase of experimental autoimmune encephalomyelitis (EAE), an animal model of the chronic inflammatory demyelinating disease MS, CD31-positive blood vessels become intensely SPARC positive by immunohistochemistry (Roskams Lab, unpublished observations). Extending beyond the vasculature to glial cells in close proximity, another study demonstrated that up-regulation of SPARC secreted by IFN-γ-treated astrocytes induced apoptosis of myelin-specific autoreactive CD4+ T cells that otherwise mediate the pathogenesis of EAE and MS, suggesting a role for SPARC in protecting the CNS against autoimmune-mediated damage during the course of neuroinflammation (Hara et al.). Furthermore, increased bioavailability of SPARC at the neurovascular niche may promote CNS regeneration by supporting neurite outgrowth after neuronal injury [34].

Although the role of SPARC at sites of injury remains to be fully characterized, experimental evidence suggests SPARC promotes the effector phase of inflammation by facilitating leukocyte transmigration. A study using HUVEC and primary lung and cardiac endothelial cell cultures demonstrated that SPARC is a VCAM1 (vascular cell adhesion molecule) ligand that induces cytoarchitectural rearrangement and intercellular gap formation, two processes critical for leukocyte trafficking [38]. In SPARC-null mice, inflammatory recruitment of neutrophils, eosinophils, and monocytes/macrophages to an inflamed peritoneum was compromised compared to those of the wildtype [38]. To that effect, SPARC-null mice immunized with myelin oligodendrocyte glycoprotein (MOG_35–55_) exhibited delayed onset and reduced severity of EAE, consistent with milder demyelination and immune infiltration, as observed by histopathological examination [51]. These findings support the argument that regulation of SPARC expression may play a key role in the mediation of inflammation through its influence on immune trafficking into inflamed tissue.

This study reports that two inflammatory molecules, TNF-α and LPS, increase SPARC expression in human cerebral endothelial cells enriching SPARC bioavailability at the neurovascular niche. Astrocyte-derived SPARC was recently shown to specifically antagonize the synaptogenic effect of SPARC-like 1 and decrease the number of axosomatic synapses in contact with lumbar spinal motor neurons, worsening the severity of paralysis during EAE in mice [52]. In both remitting and non-remitting EAE models, SPARC-like 1/SPARC mRNA and protein ratios inversely correlated with clinical paralysis severity scores. Decreased SPARC-like 1/SPARC ratios also corresponded with diminished expression of presynaptic and postsynaptic proteins as well as the number of axosomatic synaptic contacts. In vitro experiments using cortical astrocytes further revealed that T cell derived pro-inflammatory cytokines including TNF-α and IL-17 inhibit SPARC-like 1 and promote SPARC expression, favoring synaptic retraction, while anti-inflammatory cytokines such as IL-10 did the opposite thus shifting the ratio towards synaptic stabilization [52]. These data taken together support a model whereby SPARC up-regulation by pro-inflammatory cytokines drives motor synaptic disassembly, contributing to neurological deficits during the course of neuroinflammation. Indeed, our study provides in vitro evidence that cerebral endothelial cells are an abundant source of SPARC at the neurovascular niche when exposed to pro-inflammatory cytokines or bacterial endotoxin. During development, elimination of excess synaptic contacts is thought to refine circuit function [53]; however, whether this is an adaptive or maladaptive response in the context of CNS inflammation is unclear. It is thus reasonable to contend that regulation of endothelial-derived SPARC under inflammatory conditions may contribute to transient paralysis and neurological dysfunction by destabilizing synapses in the presence of neuroinflammation.

### SPARC regulation of BBB integrity and permeability

As BBB disruption is among the earliest events in several CNS pathologies such as MS, we examined the influence of SPARC on barrier function and TJ expression using an in vitro BBB model. The involvement of SPARC in the development, maintenance, and breakdown of blood-neural barriers is implicated by its spatiotemporal localization at the BBB and blood-cerebral spinal fluid barrier (BCSFB) during development and inflammation. Ependymal cells of the BCSFB lining brain ventricles and the choroid plexus express SPARC during embryogenesis and postnatal development but limit expression with maturity to barrier sites of the lateral ventricle in adult mice [23]. Analogously, brain endothelial cells of developing blood vessels arising from the pia mater strongly express SPARC during embryogenesis but down-regulate it during early postnatal development as they mature and establish a BBB [22]. In adult mice, SPARC is concentrated in mature astrocytic endfeet of the BBB [23]. Astrocytes and the soluble mediators they produce can up-regulate TJ and transporter expression on brain endothelia and are thought to be major regulators of BBB development [2, 54]. The localization of SPARC to astrocytic endfeet and cerebral endothelia place SPARC in an ideal niche to induce, maintain, or disrupt blood-neural barrier characteristics [11]. SPARC in astrocytic endfeet could reflect an uptake of endothelial-derived SPARC, an accumulation of astrocyte-derived SPARC into its end feet, or both.

TJ protein levels were quantified in lysates from confluent hCMEC/D3 cultures treated for 24 h with various doses of rh-SPARC or rhTNF-α. The reduction of TJ protein expression provides the first documentation of SPARC regulation of TJ modulation in endothelial cells. An important initial consideration, here, is the physiological relevance of SPARC doses used. In normal healthy individuals, cerebral endothelial cells reside in plasma concentrations of SPARC between 0.1 and 0.8 μg/ml, while increased concentrations have been associated with pathological conditions such as neoplasia, trauma, heart, and kidney diseases [25, 27, 30, 31]. In our experiments, elevated SPARC concentrations, modeling that expected during inflammation, lowered ZO-1 and occludin expression, suggesting that SPARC may indeed be involved in mediating TJ loss and associated BBB breakdown at supraphysiological levels. Functional assays performed in parallel showed that increasing concentrations of recombinant human SPARC applied exogenously increased transendothelial permeability of cell monolayers as evidenced by increased dextran diffusion and decreased TEER impedence. TNF-α applied exogenously as a positive control decreased total expression of all TJ proteins studied, consistent with previous reports in the literature [4, 8, 14]. Hydrocortisone offered some protection against the reduction of ZO-1 but not occludin. This may be explained by a greater ability of hydrocortisone to drive ZO-1 expression than occludin in hCMEC/D3 cultures [4].

The prevailing understanding from these experiments was that SPARC increases transendothelial permeability and disrupts endothelial barrier function in vitro through its effect on the relative state of endothelial differentiation. SPARC has also been shown to modulate transendothelial permeability through protein tyrosine kinase (PTK) phosphorylation signaling. The potent PTK inhibitor, herbimycin A, diminished SPARC-induced changes in permeability. A marked 12-fold increase in phosphotyrosine-containing proteins was immunolocalized to interendothelial borders within 1 h of SPARC treatment concomitant to barrier opening as evidenced by immunocytochemistry and BSA diffusion permeability assay, respectively. These findings are consistent with our data demonstrating decreased TJ expression and increased barrier permeability in SPARC-treated hCMEC/D3 monolayers, suggesting a putative role for SPARC in neurological conditions such as MS, characterized by microvascular TJ loss and barrier disruption [10]. Clearly, SPARC has the potential to influence BBB integrity, but the exact nature and outcome of this influence—be it reparative or pathological—is yet to be determined.

In the present study, SPARC concentrations expected during angiogenesis, inflammation, and wound repair lowered ZO-1 and occludin expression and increased endothelial barrier permeability. TJ depletion is one mechanism conventionally associated with BBB breakdown in diseases such as MS. VEGF-induced BBB breakdown is associated with occludin down-regulation and severe disability in EAE [9]. Transient global cerebral ischemia diminished SPARC mRNA expression and depleted SPARC in the basement membranes of laser-captured microdissected (LCM) blood vessels after 24 h of reperfusion [7]. Functional assays showed that the post-ischemic cerebral microvessels were more permeable than the control vessels. Here, mild post-ischemic hypothermia (~33 °C) attenuated loss of SPARC content in the basement membrane and protected against BBB breakdown, suggesting that the release of SPARC from the basement membrane was responsible for barrier opening. In this setting, SPARC released from the basement membrane after ischemic injury could act in an autocrine/paracrine manner and drive barrier disruption and vessel leakiness through endothelial VCAM1 signaling. In a recent article, tumor-derived SPARC was found to trigger endothelial paracellular permeability in primary human umbilical vein endothelial cell (HUVEC) monolayers via VCAM1 and p38 signaling, thus disrupting endothelial monolayer integrity [55]. Taken together, our study establishes SPARC as a novel regulator of paracellular cerebrovascular permeability and TJ expression, revealing a targetable interaction in CNS pathology for prevention of barrier disruption.

## Conclusions

In summary, we describe elevated SPARC expression in cerebral endothelial cells under developmental conditions modeling angiogenesis and differential regulation in the presence of inflammatory cytokines TNF-α, IFN-γ, and the bacterial endotoxin LPS. Exposure to SPARC at levels greater than those observed under normal physiological conditions increases barrier permeability and decreases TJ expression of ZO-1 and occludin. Microvascular endothelial cell exposure to SPARC may contribute to pathological processes in the CNS or facilitate its response to injury. Our study defines SPARC as an attractive target for further scientific inquiry with regard to its role in both pathological and potentially reparative processes at the neurovascular niche.

## Additional file

Additional file 1: Figure S1. Subconfluent hCMEC/D3 regions exhibit more intense SPARC expression than confluent regions by regional immunocytochemistry analysis. SPARC intensity was measured in mean pixel intensity (MPI ± SD) for selected cell-covered regions. SPARC staining in the subconfluent cultures (3.85 ± 0.49, *n* = 21 images, 7 from each triplicate well) was greater than that in the confluent cultures (2.83 ± 0.43, *n* = 14 images, 7 from each duplicate well). Bars represent the average of results from *n* = 21 and *n* = 14 images, respectively. Error bars represent SD. Data were analyzed for regional SPARC MPI in one experiment. Mann-Whitney comparison test, ****P* < 0.0001. (TIF 40 kb)

## Abbreviations

BBB: Blood-brain barrier
CAM: Chorioallantoic membrane
CNS: Central nervous system
hCMEC/D3: Human cerebral microvascular endothelial cell
IFN-γ: Interferon gamma
LPS: Lipopolysaccharide
MS: Multiple sclerosis
RT-PCR: Reverse transcription polymerase chain reaction
SPARC: Secreted protein acidic and rich in cysteine
TGF-β: Transforming growth factor beta
TJ: Tight junctions
TNF-α: Tumor necrosis factor alpha
VCAM1: Vascular cell adhesion molecule 1
VEGF: Vascular endothelial growth factor
ZO-1: Zonula occludens
