# Supplementary figures and images for: SPARC expression by cerebral microvascular endothelial cells in vitro and its influence on blood-brain barrier properties
Source: J Neuroinflammation. 2016 Aug 31;13:225. doi: 10.1186/s12974-016-0657-9 (PMC5007716; doi:10.1186/s12974-016-0657-9)

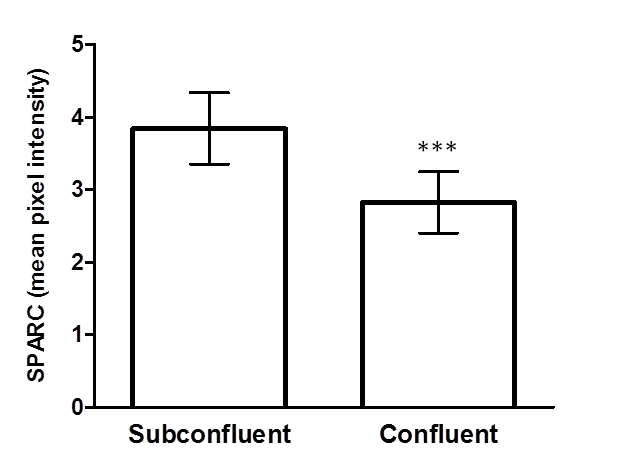

Supplement: Additional file 1: Figure S1. — Subconfluent hCMEC/D3 regions exhibit more intense SPARC expression than confluent regions by regional immunocytochemistry analysis. SPARC intensity was measured in mean pixel intensity (MPI ± SD) for selected cell-covered regions. SPARC staining in the subconfluent cultures (3.85 ± 0.49, n = 21 images, 7 from each triplicate well) was greater than that in the confluent cultures (2.83 ± 0.43, n = 14 images, 7 from each duplicate well). Bars represent the average of results from n = 21 and n = 14 images, respectively. Error bars represent SD. Data were analyzed for regional SPARC MPI in one experiment. Mann-Whitney comparison test, ***P < 0.0001. (TIF 40 kb) [file 12974_2016_657_MOESM1_ESM.tif]
